# Progress of Application Researches of Porous Fiber Metals

**DOI:** 10.3390/ma4040816

**Published:** 2011-04-19

**Authors:** Zhengping Xi, Jilei Zhu, Huiping Tang, Qingbo Ao, Hao Zhi, Jianyong Wang, Cheng Li

**Affiliations:** 1 State Key Laboratory of Porous Metal Materials, Northwest Institute for Non-ferrous Metal Research, Xi’an 710016, China; E-Mails: xizp@c-nin.com (Z.X.); hptang@c-nin.com (H.T.); aqbpshi@yahoo.cn (Q.A.); zhihao1983@163.com (H.Z.); wangjy73@163.com (J.W.); etoleecheng@yahoo.com.cn (C.L.); 2 State Key Laboratory for Mechanical Behavior of Materials, Xi’an Jiaotong University, Xi’an 710049, China

**Keywords:** metal fiber, porous material, sound absorption, heat transfer, energy absorption

## Abstract

Metal fiber porous materials with intrinsic properties of metal and functional properties of porous materials have received a great deal of attention in the fundamental research and industry applications. With developments of the preparation technologies and industrial requirements, porous fiber metals with excellent properties are developed and applied in many industry areas, e.g., sound absorption, heat transfer, energy absorption and lightweight structures. The applied research progress of the metal fiber porous materials in such application areas based on the recent work in our group was reviewed in this paper.

## 1. Introduction

Metal fiber porous materials has received much attention due to their unique controllable porous structure and special physical properties, such as lower density, larger specific surface area, higher mechanical strength and excellent permeability, and show good potential for applications in the industries, since they combine the properties of metal and its internal porous structure. With the increasing requirements of industry, metal fiber has shown its superiority in a very wide application area, e.g., textile, filtering, sound absorption, heat transfer, battery electrodes and fiber reinforced composites. In the metal fiber family, many types have been developed, e.g., stainless steel fiber, nickel fiber, aluminum and its alloy fiber, and iron inter metallic fiber, *etc.* The metal fibers can be prepared by four methods: high-temperature spraying molten metal (melt spinning method), cutting method, drawing method, and the chemical method. Normally, the diameter of the fiber obtained by these four techniques is in the range of 1~100 μm.

As a new generation of the metal porous material, metal fiber porous material contains a large number of irregular pores constructed by the fiber and pore geometries. Metal fibers cross each other and are packed together like a bird’s nest. In the traditional application areas, such as filtration and separation, metal fiber porous material becomes the most popular product because of its good properties, e.g., permeability, high strength, corrosion resistance, high temperature resistance, foldable, renewable, and long service life, *etc*. The pore structures can be controlled for high filter accuracy and greater dirt holding capacity than mesh and sintered powder filter (see Table 1) [1].

**Table 1:** Performance comparison between the sintered metal fiber felt and other filter materials.

	Compared with mesh	Compared with sintered powder filter
Dirt-holding Capacity	>3~4 times	>1.5~5 times
Air Permeability	>2~3 times	>21~600 times
Filtration efficiency	>3~15 times	>2~5 times
Porosity	>4~20 times	>2~10 times

Due to its high efficiency in applications, metal fiber porous material can be applied under special conditions with high temperature, high pressure and corrosive environment. Metal fiber porous materials used for filtration are usually prepared to be about a 1–2 mm thickness felt made of stainless steel fibers and FeCrAl fibers. They are widely used in polymers filtration, food and beverage filtration, hot gas filtration, automobile airbags, and so on. For instance, gradient filter materials made from metal fiber possess a higher filtering accuracy than other filter materials used in juice filtration; used as filter parts for vehicle safety air bags, it can control the gas expansion velocity after impact, filter the particles in the gas at high temperature and cool down the hot gas to protect the body in a form of airbag [2].

The porosity of metal fiber porous material can be as high as 98% while the pore size is smaller than 10 µm, and the three-dimensional pore space is constructed based on the inter-connections. Fiber porous metals with high porosity and small pore size are features of interest due to their typical integration structure and the functional material. This kind of metal porous material attracts engineers’ attention and stimulates researches of materials science. The special and designable properties of the pore structure for the metal fiber porous materials provide a wide application in many industrial areas. The research progress regarding the porous metal fiber material in the application of sound absorption, enhanced phase change heat transfer, energy absorption is reviewed in this paper based on research work of authors’ group.

## 2. Progress of Application Researches of Sound Absorption

Compared with mineral wool, wood fiber board and polymer foam, the metal fiber porous material shows a great potential in the properties of sound absorption, high strength, high temperature resistance, anticorrosion, air impact resistance, weather resistance and designable structure [3]. The metal fiber porous material is becoming irreplaceable for noise-control in harsh environments. For instance, fiber porous sound-absorbed materials, prepared by 1Cr18Ni9 stainless steel fibers of diameter 8~100 μm, were applied by Boeing as silencer in intake, exhaust port auxiliary unit of Aero-engine and sound absorption liner in the engine. Absorption coefficient of this kind of fiber porous material is close to the ultra-fine glass wool with 750 Hz, higher than the ultra-fine glass wool above 750 Hz. Aluminum fiber absorbing materials have been used in noise control of concert halls, exhibition halls, classrooms, and highways, subways, tunnels and the other humid underground areas. Recently, metal fiber materials have been applied as silencers in cars, such as Audi and Santana [4].

Generally, the sound absorption properties of porous material are good in high-frequency but poor in low frequency based on the sound adsorbing principle. By designing and optimizing the pore structure, the sound absorbing performance of the fiber porous material is significantly improved in low frequency and the bandwidth of the sound adsorption. Wang X.L. reported on a semi-empirical and nonlinear flow resistance model for metal fiber materials [5,6]. This model combined with the acoustic model of Umnova-Attenborough can predict the acoustic performances of the metal fiber porous materials. Zhang Bo proposed a relatively simple extended model of sound absorption properties for the metal porous materials [7].

**Figure 1:**
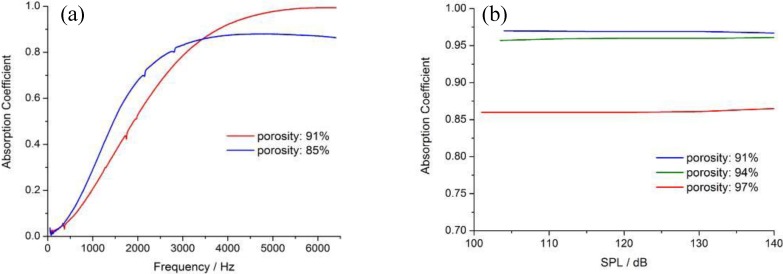
Sound absorption properties of FeCrAl fiber porous material: (**a**) the influence of porosity; (**b**) stable properties at different SPL.

Tang H.P characterized the sound absorption properties of the metal fiber porous materials with different structures to optimize pore structure, and investigate the effects of the porosity, thickness of material and airspace on sound absorption performances [8,9,10]. The metal fiber porous materials prepared show excellent sound performances. The acoustic absorption coefficient of FeCrAl fiber porous materials of 91% porosity and 10 mm thickness is more than 95% in the frequency range from 4,500 to 6,400 Hz (shown in Figure 1). It was found that the porosity and the diameter play a significant role in sound absorption. The sound absorption shows a good performance in the medium and low frequency range with a smaller porosity, greater thickness and larger diameter, but it performs poorly in the high frequency region. Nevertheless, the FeCrAl fiber porous material maintains a stable absorption property in a wide range of sound pressures varying from 100 dB to 140 dB, as shown in Figure 1(b). Effect of high intensity sound on sound adsorption performance of gradient structure was similar to ordinary sound pressure from 20 dB to high sound intensity conditions of 100 dB. Other structures do not have this characteristic, such as perforation plates.

**Figure 2:**
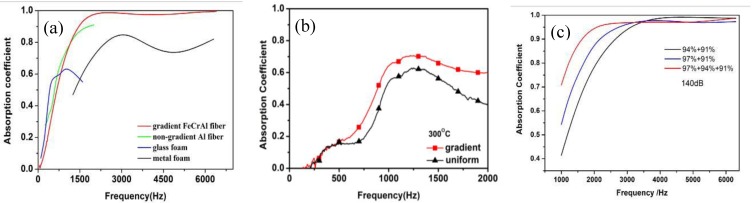
Sound absorption properties of gradient under different conditions: (**a**) normal conditions; (**b**) at high temperature; (**c**) at high intensity pressure.

The metal fiber porous material with a gradient pore structure was prepared to improve sound absorption properties. As shown in Figure 2, the optimized gradient fiber porous materials have excellent sound absorption properties, even at high temperature and in high intensity conditions. The sound absorption performance at low frequency has been significantly improved while the sound absorption coefficient at high frequency remains at a high position (Absorption-frequency curve is flat). The absorption properties changes as the porosity varies from the highest to the lowest in gradient porous structures. Therefore, it can be concluded that the gradient structure has an important effect on the absorption properties. It was found that the gradient porous structure with a higher porosity and greater thickness presents better sound absorption.

## 3. Progress of Application Researches of Phase Change Heat Transfer

Phase change heat transfer as a kind of advanced heat transfer has been used in many fields. Metal fiber porous material is a perfect working media where phase change heat transfer occurs [11], which plays a significant role in numerous industrial applications because of high boiling heat transfer, low boiling temperature difference, good blockage resistance, long life, *etc*.

Pore structure is the key factor to the heat transfer properties of fiber porous material. In the early 1980s, AG Kostornov and LG Galstyanfrom made systematic studies on the influence factors of heat transfer coefficient of metal fibers [12], and indicated that the thermal conductivity of the material drops with an increase in fiber diameter, a significant decrease in thermal conductivity is promoted by another factor having a detrimental effect on the quality of the contacts between the fibers, a drop in sintering temperature. L. Tadrist analyzed in detail the effects of the fluid flow rate of liquid in porous surface and aspect ratio of metal fiber on heat transfer performance under forced convection without phase change [13]. The studies from South China University of Technology show that porosity is one of most important structural parameters influencing heat transfer performance, especially in the low filling rate [14].

**Figure 3:**
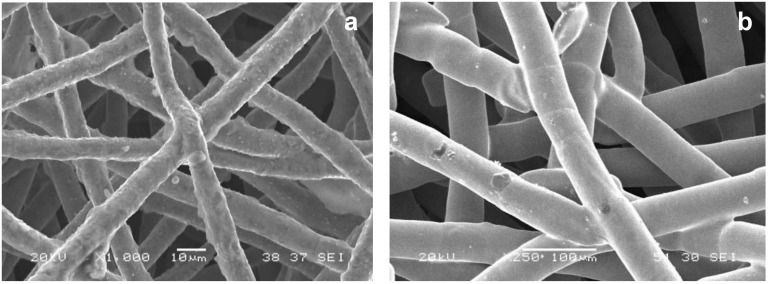
SEM photos of sintered porous materials with stainless steel fibers (**a**); and copper fibers (**b**).

**Figure 4:**
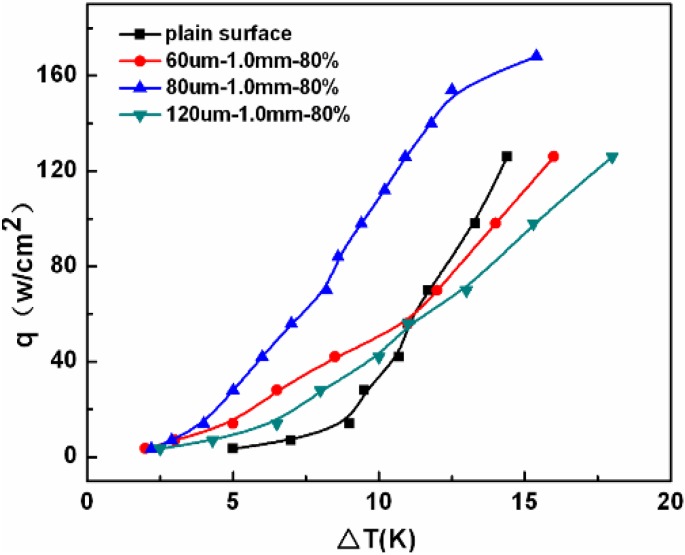
The boiling curves of metal fiber porous surface.

Authors of this paper prepared metal fiber porous surface materials on the surface of tube and plate by sintering method [15,16]. The metal fiber porous surface materials with interpenetrating pore structures composited of randomly stacked fibers have large numbers of form metallurgical bonding from fiber to fiber and fibers to substrate (as shown in Figure 3). Such a pore structure provides numerous bubble nucleations, and ensures rapid heat transfer from substrate to porous surface, which contributes to a good heat transfer performance. The pool boiling heat transfer tests indicate that the porosity, thickness and fiber diameter of materials have great effects on pool boiling heat transfer performance [17,18]. In order to obtain optimum heat transfer enhancement effect, it is necessary to chose suitable fiber diameter, thickness and porosity. The heat transfer coefficient of porous copper fibers with optimized pore structure is about six times higher than that of smooth surface as shown in Figure 4 [9].

## 4. Progress of Application Researches of Energy Absorption and Ultra-Light Structure

With developments in aerospace, high-speed rail trains and the car industry, the sandwich structure with ultra-light material has gradually become a research focus. Sandwich structure combines many properties: ultra-light weight, high specific strength and specific stiffness, high energy absorption, sound absorption, and thermal insulation, which make it a perfect application in high energy consumption equipment (automotive, high-speed train, aerospace, ships, *etc.*) [19]. The core materials of the sandwich structure are mainly fiber material, grid material, aluminum honeycomb and aluminum foam. Theoretical and experimental studies show that porous metals have outstanding energy absorption properties. Compared with aluminum foams, metal fiber porous materials have excellent mechanical properties, such as higher compressive strength and excellent energy absorption capacity [20]. At present, there is an increasing interest being shown in the energy absorption capacity of porous metals.

Volvo Car Corporation is the first institute to research and apply metal fiber porous sandwich structure, which developed the ultra-light stainless steel sheet used sandwich structure (HSSA, Hybrid Stainless Steel Assembly) [21]. The HSSA structure is composed of stainless steel fibers (fiber length is 1 mm, diameter less than 20 μm) bonded with thin stainless steel faceplates, the HSSA is lighter and has higher rigidity than aluminum, and has characteristics of sound insulation and shock absorption. The weight of the car manufactured with HSSA is about 50%–70% lighter than traditional cars. Further research indicated that energy absorption capacity of HSSA is 50%–60% higher than solid metal plates [22,23,24]. Researches performed by Qiao J.C. show that metal fiber porous material has a strong energy absorption capacity [25]. The energy absorption capacity of the sintered metal fiber porous material is about 10 times that of aluminum foam at the same porosity. Pore structures of metal fiber porous material is the key factor that affects its mechanics and energy absorption properties. The mechanical properties of metal fiber porous material are dependent on the combination between the fibers and the number of the metallurgy node. The higher the sintering bonding per unit volume and the bonding intensity are, the better the mechanical properties of materials.

**Figure 5:**
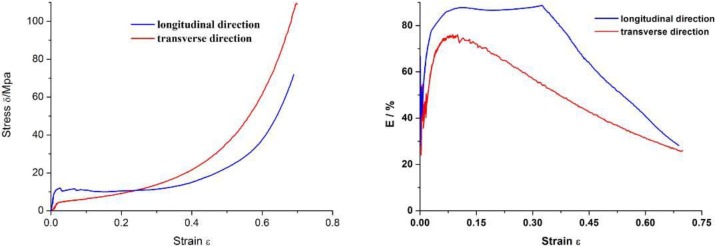
Compression anisotropy of porous metal fibers with a porosity of 80%: (**a**) Strain-stress curve; (**b**) Energy absorption efficiency.

In the preparation of metal fiber sandwich material research, Massachusetts Institute of Technology and Cambridge have developed two kinds of sandwich preparation methods: CAMBOSS (Cambridge Bonded Steel Sheets) and CAMBRASS (Cambridge Brazed Steel Sheets). The difference between CAMBOSS and CAMBRASS is the combination method of plates and metal fiber material, the former uses bonding method and the latter uses brazing method [26]. Fraunhofer-Gesellschaft in Germany prepared the aluminum fibers sandwiches by low temperature transient liquid phase sintering [27]. Cheng Li and Wang J.Y proposed and implemented porous metal fiber sandwich materials with the erect fiber core body by sintering process [28]. According to dynamic mechanical analysis, this type of fiber sandwich material is a kind of sensitive material, and its pore structure and mechanical properties of such erect fiber core body is anisotropic. As shown in Figure 5, the compression properties and energy absorption efficiency are anisotropy. There are more apparent yield wave peaks, yield wave valley and longer plastic deformation platform in the longitudinal direction, and both the Young’s module and yield strength of the porous metal fibers compressed in longitudinal direction are higher than compressed in transverse direction. The energy absorption efficiency compressed in longitudinal direction is about 25% higher than that compressed in transverse direction obviously.

## 5. Recommendations

With the progress and development of industry and technology, some new properties and applications for metal fiber porous materials are being developed. Currently, a small portion of performance and application have captured researcher’s interest, yet much more potentially could be developed or is still restricted at the experimental research stage. A lot of work is still required, focusing on preparation and application research.
